# The Arp8 and Arp4 module acts as a DNA sensor controlling INO80 chromatin remodeling

**DOI:** 10.1038/s41467-018-05710-7

**Published:** 2018-08-17

**Authors:** Sandipan Brahma, Mzwanele Ngubo, Somnath Paul, Maheshi Udugama, Blaine Bartholomew

**Affiliations:** 1 Department of Epigenetics & Molecular Carcinogenesis, Science Park, The University of Texas MD Anderson Cancer Center, Smithville, TX 78957 USA; 2 Center for Cancer Epigenetics, MD Anderson Cancer Center, Smithville, TX 78957 USA; 3 Present Address: Department of Biochemistry and Molecular Biology, Southern Illinois University, 1245 Lincoln Drive, Carbondale 62901 USA; 4 Basic Sciences Division, Fred Hutchinson Cancer Research Center, Seattle, WA 98109 USA; 5 Present Address: Department of Biochemistry and Molecular Biology, Monash University, Clayton, Vic 3800 Australia

## Abstract

Nuclear actin and actin-related proteins (Arps) are key components of chromatin remodeling and modifying complexes. Although Arps are essential for the functions of chromatin remodelers, their specific roles and mechanisms are unclear. Here we define the nucleosome binding interfaces and functions of the evolutionarily conserved Arps in the yeast INO80 chromatin remodeling complex. We show that the N-terminus of Arp8, C-terminus of Arp4 and the HSA domain of Ino80 bind extranucleosomal DNA 37–51 base pairs from the edge of nucleosomes and function as a DNA-length sensor that regulates nucleosome sliding by INO80. Disruption of Arp8 and Arp4 binding to DNA uncouples ATP hydrolysis from nucleosome mobilization by disengaging Arp5 from the acidic patch on histone H2A-H2B and the Ino80-ATPase domain from the Super-helical Location (SHL) -6 of nucleosomes. Our data suggest a functional interplay between INO80’s Arp8-Arp4-actin and Arp5 modules in sensing the DNA length separating nucleosomes and regulating nucleosome positioning.

## Abstract (web summary)

INO80 chromatin remodeler plays important roles in transcription, DNA repair, recombination, and heterochromatin maintenance. Here the authors characterise the functional interplay between the actin-related proteins (Arps)  in INO80, showing that Arp8 and Arp4 module sense the DNA length separating nucleosomes and regulate nucleosome remodeling.

## Introduction

The ATP-dependent chromatin remodeling complex INO80 has important roles in transcription, heterochromatin maintenance, DNA replication, repair, and recombination. These functions reflect the ability of INO80 to shape promoter nucleosome landscape by exchanging histone H2A.Z for H2A in nucleosomes and changing nucleosome positions^1,2^. Disruption of INO80 function is associated with cancers^3–5^ as well as coronary^6^ and other vascular diseases^7^. Recently, the structures of fungal and human INO80 complexes bound to nucleosomes were solved using high-resolution cryo-electron microscopy^8,9^. However, these structures did not define the binding interfaces of the evolutionarily conserved nuclear actin and actin-related proteins (Arps) of INO80 or provide insights into their functional properties^10–13^. Arp4 and actin are shared components of three different chromatin remodeling complexes that mechanically move or alter the composition of nucleosomes in an ATP-dependent manner (INO80 and SWR1) or post-translationally modify histones (NuA4). A similar and less conserved module comprised of Arp7 and 9 is also present in the yeast SWI/SNF and RSC chromatin remodelers. In the INO80 complex, the actin-Arp4 module has an additional Arp subunit, i.e., Arp8. When Arp8 is deleted actin and Arp4 are also lost from the complex, thereby causing the complex to lose its ability to bind DNA^11^. Arp8 is further shown to be associated with DNA by ChIP-exo in which Arp8 is broadly crosslinked with formaldehyde to linker and nucleosomal DNA^14^. The Arp8-Arp4-actin module binds to the helicase/SANT-associated (HSA) domain of the Ino80 subunit^12^. The crystal structures of the actin-Arp4-HSA(Swr1) trimer and the actin-Arp4-HSA(Ino80)-Arp8 tetramer have been solved, and mutational analysis have shown that the HSA domain binds DNA^15^ (Knoll et al. in preparation). Although these studies point to the Arp8 module binding DNA, whether this module acts to recruit INO80 to nucleosomes or has a more specific mechanistic role within INO80, is not clear. Additionally, recombinant Arp8 has been shown in vitro to bind with histones^11,16^.

Here we describe that in addition to the HSA domain Arp8 and Arp4 bind extranucleosomal/linker DNA at a discrete distance from the entry site of nucleosomes and function as a DNA-length sensor to allosterically regulate INO80-mediated nucleosome spacing. In the crystal structure of the actin-Arp4-HSA-Arp8 tetramer (Knoll et al. in preparation), Arp8 is missing its N-terminus from residues 1–254, and our data shows this missing region associates with DNA and is required for the Arp8-Arp4-actin module to bind linker DNA. Binding of the INO80 Arp8 module to linker DNA is required to couple ATP hydrolysis to nucleosome repositioning and for the proper docking of the Ino80 catalytic subunit and Arp5 to nucleosomes.

## Results

### The Arp8 and Nhp10 modules and Ino80 HSA bind linker DNA

**Fig. 1:**
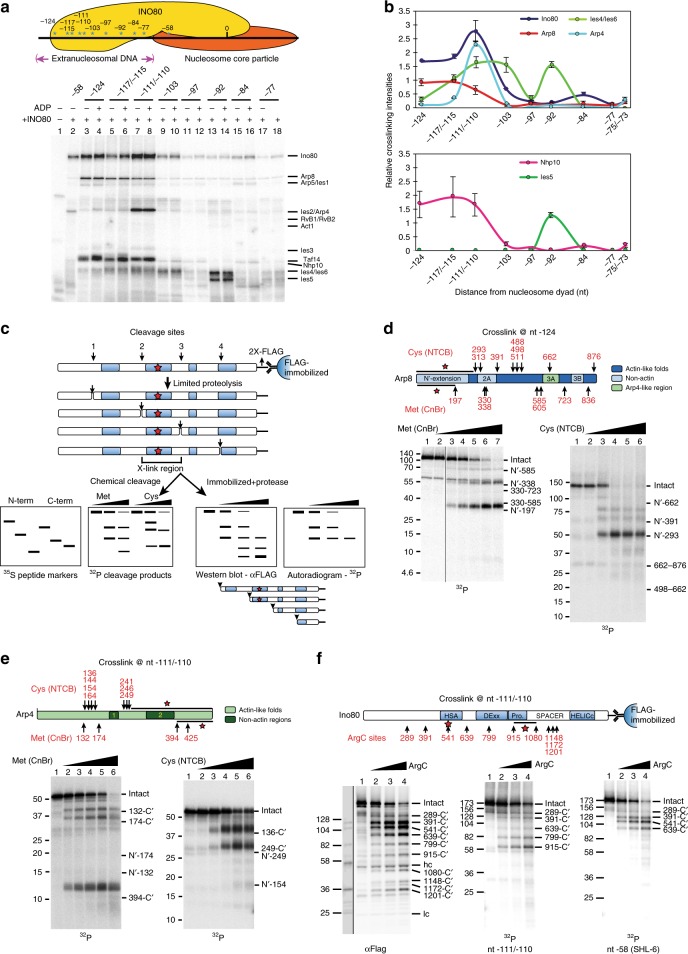
The N-terminus of Arp8, C-terminus of Arp4, and the HSA domain of Ino80 each associate with extranucleosomal DNA. **a** Top panel: Cartoon representation of the INO80 complex on nucleosomes of the sites where photoreactive nucleotides are incorporated by their distance in nucleotides from the dyad axis. Bottom panel shows photo-crosslinked INO80 subunits at each site on extranucleosomal DNA. **b** Crosslinking efficiencies of INO80 subunits at various nucleotide positions. Values are mean of ≥3 experiments, normalized to Ino80 at nt −58 without ADP. Error bars denote ± s.d. **c** Schematic outlining the protein-DNA crosslinking/peptide cleavage strategy used in d-f. Photo-crosslinked [^32^P]-radiolabeled peptides resulting from cleavage at methionines (Met) or cysteines (Cys) are compared with custom-synthesized [^35^S] peptide molecular weight markers. For photo-crosslinked Ino80 immobilized via the C-terminal FLAG and cleaved at arginines (ArgC protease), all FLAG-tagged fragments are identified by immunoblotting and are compared to photo-crosslinked fragments. **d**, **e** Schematics show the locations of Cys and Met in Arp8 (**d**) and Arp4 (**e**) plus the conserved actin-fold regions, non-actin insertions, and the regions that crosslink to DNA (highlighted by a black bar and red asterisk). Bottom panels are representative phosphorimages and the amount of CnBr or NTCB increases from left to right. Numbers on the left correspond to general protein molecular weight markers (in kDa), and on the right, correspond to custom-synthesized Arp8/Arp4 peptide markers, shown by their length in amino acids relative to the intact protein. **f** The region of Ino80 crosslinked to nt −111/−110 was mapped using ArgC protease. Cleavage sites are depicted as in **d** and **e**. Numbers on the left correspond to custom-synthesized markers (their molecular weights expressed in kDa), and on the right, indicate the lengths of the peptides resulting from ArgC cleavage, in number of amino acids from the C-terminus. Uncropped images showing the peptide-mapping fragments alongside custom-synthesized markers on the same gel are shown in Supplementary Fig. 2

We probed INO80 interactions at eight different positions in extranucleosomal DNA by DNA crosslinking^17,18^. The Ino80 catalytic subunit along with subunits of the Arp8 module bind to extranucleosomal DNA 110–124 nucleotides (nt −110 to −124) from the dyad axis or 37–51 base pairs (bp) from the edge of nucleosome core particles, and the yeast specific Nhp10 module binds to a wider region on extranucleosomal DNA (nt −92 to −124), 18–51 base pairs (bp) from the edge of nucleosome core particles (Fig. 1a, b). The yeast Arp8 module binds the HSA domain of Ino80 and besides the three conserved subunits contains the yeast specific Ies4 and Taf14 subunits^12,19^. Of the 5-subunit Arp8 module, we find only the Arp4 and Arp8 subunits directly associating with extranucleosomal DNA as seen by site-directed DNA crosslinking. Immunoprecipitation confirms that Arp4 is crosslinked and not Ies2, which has a similar electrophoretic mobility (Supplementary Fig. 1a). Consistent with our results ChIP-exo shows Arp8, Nhp10, and Ies5 interacting with the linker DNA associated with promoter-proximal +1 nucleosomes^14^, but rather than to a broad stretch of DNA we show that Arp8 is bound to a 14 base pair region of extranucleosomal DNA spanning from 110 to 124 bp from the dyad axis (nt -110 to -124). The Ino80 catalytic subunit associates with this same region and Arp4 to a narrower part spanning from 110 and 111 bp from the dyad axis. Chemical cleavage of photo-crosslinked Arp8 and Arp4 at methionines and cysteines reveal that the N-terminus of Arp8 (residues 1–197) associates with extranucleosomal DNA at nt−124, while the C-terminus of Arp4 (residues 425–489) near the barbed end of the actin-fold^15,20^ associates with nts −110/−111 (Fig. 1c–e and Supplementary Fig. 2a-d). The HSA domain of Ino80 (near residue 541) and the protrusion region 2/spacer (residues 915–1088) are both shown to bind at nts −110/−111 and −124 by enzymatic cleavage of photo-crosslinked Ino80 with ArgC protease and immobilization of the C-terminal fragments (Fig. 1f and Supplementary Fig. 2e). We interpret the peptide mapping as showing that two distinct sites are crosslinked, because cleavage at residue 541 appears to be blocked and the other cleavage products indicate crosslinking between residues 915 and 1080. The Ino80 HSA domain could be expected to bind the same region as Arp4 and Arp8 given the HSA domain binds and recruits the Arp8 module^10–12,19^. These data uniquely define the previously unknown DNA-binding interface of the Arp8 module to be comprised of Arp8, Arp4, the HSA domain, and the protrusion-2 region of Ino80 that bind extranucleosomal DNA more than 37 bp from the edge of nucleosomes.

We find the Nhp10 and Ies5 subunits bind to nearly the same stretch of extranucleosomal DNA as the Arp8 module in two distinct regions, at nts −110 to −124 (Nhp10) and −92 (Ies5) (Fig. 1a, b). The Nhp10 module is comprised of Nhp10, Ies1, Ies3, and Ies5, and this yeast specific subcomplex and its metazoan-specific version bind to the extreme N-terminus of Ino80^11,19,21^. The metazoan subcomplex is not required for INO80 remodeling^21,22^, whereas the yeast Nhp10 module has a role in sensing the DNA length between nucleosomes^23^. Nhp10 is an HMG-like protein previously suggested to bind linker DNA, but the precise position and nature of its interactions was not known^14,23,24^. Since Nhp10 and Taf14 have similar electrophoretic mobilities, we confirmed the identity of the crosslinked protein by observing the shift in the mobility of the crosslinked species when three copies of an epitope tag (HA, hemagluttin) are attached to the C-terminus of Nhp10 (Supplementary Fig. 1b, c). Either the Ies4 or Ies6 subunit also crosslink at nt −92 and was not differentiated at this time (Supplementary Fig. 1a).

### The Arp8 DNA-binding region is essential for INO80 functions

**Fig. 2:**
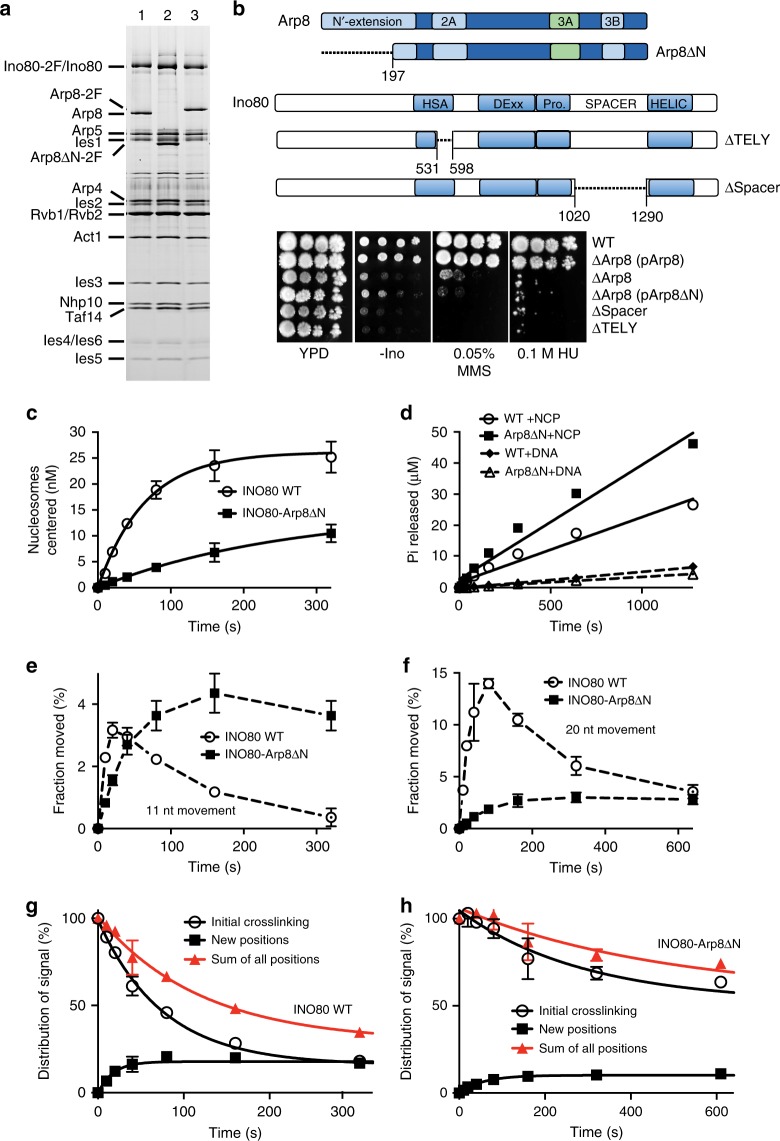
The N-terminus of Arp8 is required for the in vivo and nucleosome mobilization activities of INO80. **a** Complex integrity is maintained in purified wild-type INO80 with Ino80-2FLAG (lane 1), INO80-Arp8ΔN with 2FLAG-Arp8ΔN (lane 2), and INO80 with 2FLAG-Arp8 (lane 3). **b** Top panel: Schematic depicting deleted regions in Arp8 (Arp8ΔN) and Ino80 (ΔTELY and ΔSpacer) mutants. Plasmids expressing wild type or mutant Arp8 (Arp8∆N) were introduced into *arp8* null yeast and compared to strains lacking INO80 function (∆Spacer and ∆TELY). **c** Plots showing the rates at which wild type and Arp8ΔN INO80 move 70N0 nucleosomes (representative native electrophoretic mobility shift assay (EMSA) images shown in Supplementary Fig. 3a). **d** Plots based on thin-layer chromatography data showing the rates of ATP hydrolysis of wild type and Arp8ΔN INO80 in the presence of either nucleosomes or naked DNA assayed in the same conditions as in **c**. **e**, **f** DNA movement on the histone octamer surface of nucleosomes was measured using nucleosomes modified at residue 53 of H2B (see Supplementary Fig. 3c). Plots show the fraction of nucleosomes that moved 11 (**e**) and 20 (**f**) base pairs from the starting position versus time by wild type and Arp8ΔN INO80. **g**, **h** The amount of DNA cleaved at the starting position (initial crosslinking), the total amount of cleaved DNA that accumulates during remodeling (new positions), and sum of the intensities of the initial and remodeled positions (sum of all positions) track the retention of histone-DNA contacts versus time for wild type (**g**) and Arp8ΔN INO80 (**h**). Error bars in **c**–**h** denote s.e.m. from ≥ 3 experiments

We deleted the N-terminus of Arp8 to determine if loss of this region alone disrupts complex integrity, reduces the enzymatic activity of INO80, or binding of the Arp8 module to linker DNA. Whereas deletion of the entire Arp8 subunit causes a loss of Arp4 and actin from the INO80 complex^11^, deletion of the N-terminus of Arp8 maintains the integrity of the INO80 complex and retains Arp4, actin, and the truncated Arp8 (Fig. 2a). Despite forming a stable INO80 complex, cells expressing truncated Arp8 (Arp8ΔN-INO80) are phenotypic of cells completely lacking Arp8^11^, as they are sensitive to replication stress (HU), DNA damage (MMS), and are defective for INO80-dependent regulation of transcription (-ino) (Fig. 2b). Cells with Arp8ΔN-INO80 are also similar to those in which the highly conserved TELY motif in the Ino80 post-HSA domain from amino acids 531 to 598 (∆TELY) are deleted. Deletion of the TELY motif had been shown previously to reduce the association of actin, Arp4 and Arp8 with the rest of the INO80 complex, similar to *arp8* null^10^. We also find the Arp8ΔN-INO80 strain to behave similar to those in which the spacer (or insert) region from amino acids 1018 to 1299 between the two ATPase lobes is deleted (∆Spacer), causing the loss of the Arp5, Ies6, Ies2, and Rvb1/2 subunits and disrupting the in vivo activity of INO80^25^. Our data suggest that the biochemical activity of the Arp8ΔN-INO80 complex is distinct from that in which Arp8, Arp4 and actin are absent^11,19^, as the nucleosome binding and ATPase activities of Arp8ΔN-INO80 are not significantly reduced compared to wild-type INO80; however, nucleosome mobilization is reduced 6-fold (Fig. 2c, d, and Supplementary Fig. 3a, b). This uncoupling of ATPase activity from nucleosome mobilization indicates that the Arp8ΔN-INO80 complex retains particular contacts with nucleosomes critical to stimulate its ATPase activity and has specifically lost other key interactions required to move DNA through nucleosomes.

We examined the defects in nucleosome mobilization further by mapping changes in histone-DNA contacts as DNA tracks across the octamer surface^17,26^. We find that the Arp8ΔN complex moves the first 11 bp of DNA at nearly the same rate as wild-type INO80, indicating that it can readily complete this early step in remodeling but cannot efficiently move DNA farther (Fig. 2e, f, and Supplementary Fig. 3c). Besides moving DNA along the surface of the histone octamer, INO80 can persistently displace DNA from the surface of the H2A-H2B dimer in an ATP-dependent manner^17^. We track DNA displacement at the H2A-H2B interface by examining the sum of all INO80-remodeled products including new and starting positions and whether that sum decreases over time (Fig. 2g and Supplementary Fig. 3c lanes 1–9). The Arp8ΔN complex displaces DNA from the H2A-H2B dimer two times less efficiently than wild-type INO80 despite an increased rate of ATP hydrolysis (Fig. 2h, Supplementary Fig. 3c lanes 10–18 versus lanes 1–9, and Fig. 2d).

### Arp8-DNA interaction regulates key INO80-DNA contacts

**Fig. 3:**
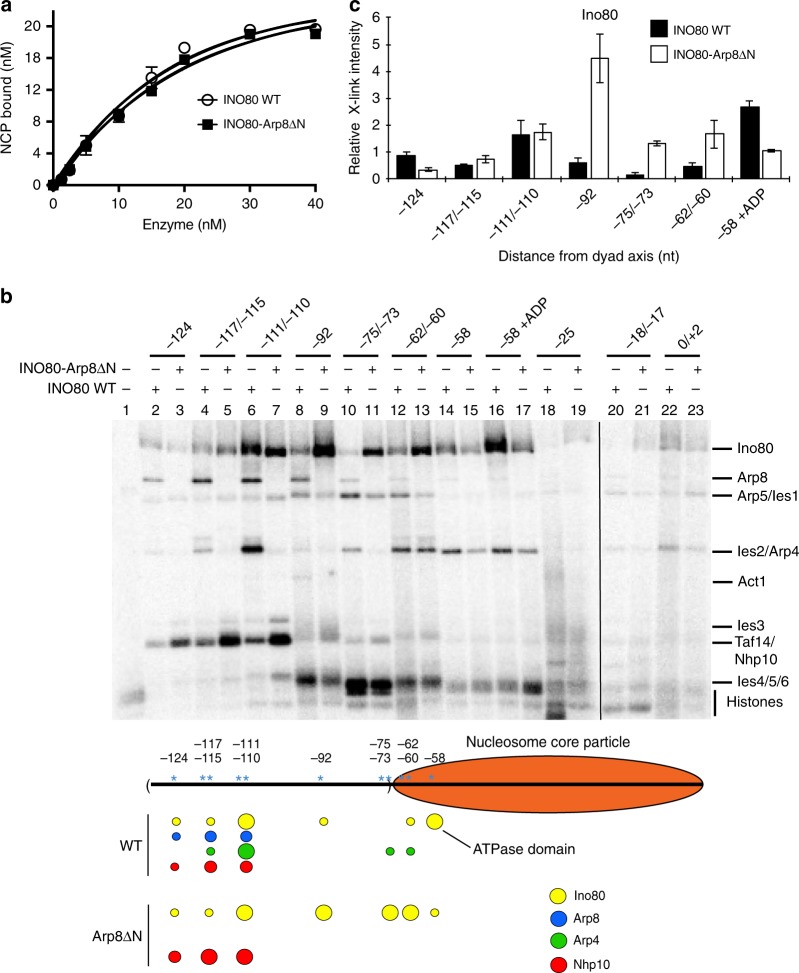
Binding of Arp8 and Arp4 to extranucleosomal DNA mediates the proper docking of the Ino80 catalytic subunit onto nucleosomes. **a** Plots showing the fractions of end-positioned (70N0) nucleosomes bound by wild type and Arp8ΔN INO80 versus increasing concentrations of the enzymes (representative native electrophoretic mobility shift assay (EMSA) images shown in Supplementary Fig. 4a). Error bars denote s.e.m. from three experiments. **b** Site-specific interactions of wild type and Arp8ΔN INO80 with nucleosomal and linker DNA were mapped by DNA crosslinking as in Fig. 1a. ADP was added in lanes 16 and 17 (Fig. 1a and ref.^17^). **c** Crosslinking efficiencies of the Ino80 catalytic subunit in wild type and Arp8ΔN INO80 (from **b**) at specified nucleotide positions. The schematic below shows the changes in Ino80, Ap8, Arp4, and Nhp10 crosslinking between wild type and Arp8ΔN INO80. Circle sizes are proportional to crosslinking efficiencies at the specified DNA positions. Values are mean of three experiments, normalized to the Ino80 subunit of wild type at nt −58 without ADP (lane 14 in panel **b**). Error bars denote ± s.d

Next, we explored why loss of the N-terminus of Arp8 uncouples nucleosome movement and DNA displacement from ATP hydrolysis by re-examining the INO80-nucleosome binding interfaces. Loss of the N-terminus of Arp8 does not reduce the affinity of INO80 complex for nucleosomes, likely reflecting the multiple interactions that are involved in INO80 binding (Fig. 3a and Supplementary Fig. 4a). DNA crosslinking confirmed that the N-terminus of Arp8 is required for stable binding of both Arp8 and Arp4 to extranucleosomal DNA (nts −124 to −75/−73) (Fig. 3b lanes 2–11 comparing odd and even lanes, and Supplementary Fig. 4b-d). We also show that attachment of a HA-tag at the C-terminus of Arp4 interferes with Arp4 and Arp8 binding at nts −110/−111, consistent with the C-terminus of Arp4 binding DNA (Supplementary Fig. 1c lanes 6 and 7). Binding of the Nhp10 subcomplex to extranucleosomal DNA is however not blocked and its crosslinking increases when the N-terminus of Arp8 is absent or the C-terminus of Arp4 is tagged (Fig. 3b lanes 2–7 plus the lower panel, Supplementary Figs. 1c, lanes 6 and 7, and 4e).

DNA crosslinking experiments revealed that stable binding of the ATPase domain of Ino80^17^ and Ies2 to DNA at the super-helical location (SHL)-6 in nucleosomes (nt −58) is reduced by deletion of the N-terminus of Arp8 (Fig. 3b lanes 14–17, Fig. 3c, and Supplementary Fig. 4d). Previously, Ies2 was shown to bind to the ATPase domain of Ino80, consistent with our DNA crosslinking data for Ies2^19^. Furthermore, Ino80 binding shifts from SHL-6/-5 to extranucleosomal DNA up to 92 nucleotides from the dyad axis (Fig. 3b lanes 8–13, compare odd and even lanes, plus the lower panel, and Fig. 3c). There are two possible reasons for these changes interfering with INO80 moving nucleosomes. First, displacement of DNA from a part of the histone octamer surface caused by the ATPase domain binding at SHL-6 is likely absent when the N-terminus of Arp8 is deleted, while this displacement may prime nucleosome movement by the wild-type complex^8,9^. Second, since Ino80 shifts to an external position outside of the core nucleosomes when Arp8 is truncated, DNA translocation near the H2A-H2B dimer required by INO80 to mobilize nucleosomes will also be missing^17^.

### Arp5 binding to H2A-H2B requires Arp8-DNA interaction

**Fig. 4:**
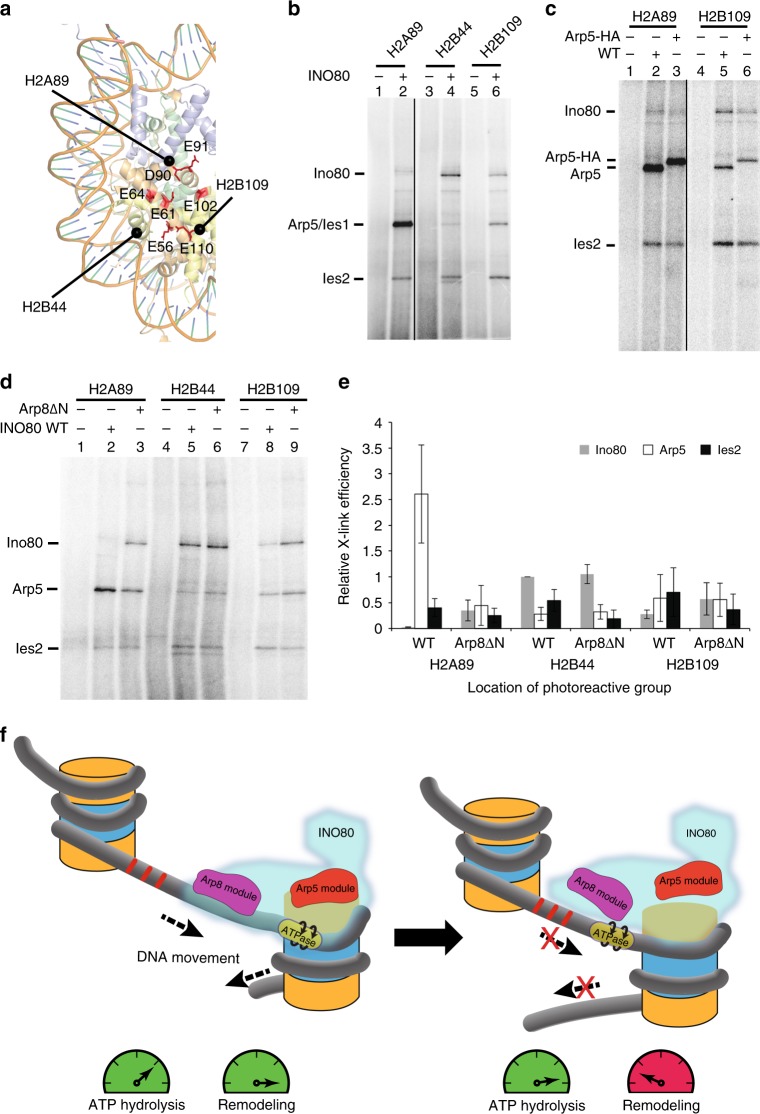
Arp5 interactions with the acidic pocket of the histones H2A-H2B dimer requires Arp8 and Arp4 binding to extranucleosomal DNA. **a** Locations of the aspartate (D) and glutamate (E) residues constituting the acidic pocket on histone H2A-H2B dimer surface are highlighted in red^31^. The sites of the modified cysteine residues used to map histone interactions of wild type and Arp8ΔN INO80 are shown as black spheres. **b**–**d** Phosphorimages of INO80 subunits tagged by crosslinking and [^125^I] radiolabel-transfer. Crosslinked Arp5, Ino80, and Ies2 subunits are indicated on the left. **c** Crosslinked Arp5 was distinguished from co-migrating Ies1 using INO80 with C-terminally HA-tagged Arp5 (Arp5-HA). **d** The crosslinking patterns of wild type and Arp8ΔN INO80 are compared. **e** Plots showing the crosslinking efficiencies of wild type and Arp8ΔN INO80 to the H2A-H2B dimer surface. Values are mean of three experiments, normalized to the Ino80 subunit of wild type at H2B44. Error bars denote ± s.d. **f** The role of the Arp8 module in regulating INO80 chromatin remodeling in a linker DNA-length dependent manner is illustrated. The Arp8 and Arp5 modules are depicted along with the ATPase domain of Ino80, all as a part of the INO80 complex. As nucleosomes are moved closer together, binding of the Arp8 module to linker DNA is disrupted, which in turn causes the Arp5 module to lift off from the histone dimer surface and the ATPase domains moves away from the core nucleosome. These conformational changes uncouple the ATP hydrolysis from the nucleosome mobilization activitiy of INO80

To further investigate changes in INO80-nucleosome interactions we probed interactions with the histone octamer surface through site-specific histone crosslinking^27^. We find that the Arp5 subunit contacts the H2A-H2B dimer near its acidic pocket, consistent with recent cryoEM structures^8,9^, and this contact is diminished when the N-terminus of Arp8 is absent (Fig. 4a, b, d, and Supplementary Fig. 5). Since Arp5 and Ies1 migrate similarly, we confirmed Arp5 photocrosslinking by comparing the electrophoretic mobility of the crosslinked species with and without HA-tagged Arp5 (Fig. 4c). The Ino80 and Ies2 subunits also bind to the H2A-H2B dimer, but to a significantly lesser extent than Arp5. Loss of the Arp5 subunit has been shown previously to uncouple the nucleosome mobilizing ability of INO80 from its ATPase activity^22,25,28^, consistent with changes we observe here with the Arp8ΔN complex. Also in alignment with our observations, interactions with the acidic pocket of H2A-H2B have been shown previously to be important for positively regulating INO80 and other chromatin remodelers^16,29–31^.

## Discussion

Taken together, our data show that Arp8 and Arp4 associate with extranucleosomal/linker DNA 37–51 bp from the edge of nucleosomes to properly orient INO80 on the nucleosomes for remodeling. These interactions promote proper docking of the ATPase domain of Ino80 inside of nucleosomes, as well as stable binding of Arp5 to the histone H2A-H2B acidic-pocket region (Fig. 4f). The loss of Arp5 contacts with nucleosomes and subsequent loss of coupling of ATP hydrolysis to nucleosome movement is consistent with the prior model of Arp5 being required as a histone and DNA anchor to stabilize Ino80 translocation on DNA and provide sufficient torque to displace and move DNA on the surface of the histone octamer^8,9^. The role of the Arp8 module binding to DNA is therefore not simply to recruit INO80 to nucleosomes as the affinity of INO80 for nucleosomes do not depend on Arp8 binding to linker DNA, but rather acting as a DNA sensor to effect allosteric changes in INO80 required for it to efficiently remodel nucleosomes. The placement of the Arp8 module onto linker DNA is in concordance with the preferred length of linker DNA required to mobilize nucleosomes and the observed nucleosome spacing activity of INO80^32^. It appears unlikely that Arp8 promotes nucleosome movement by pushing DNA into nucleosomes as DNA gaps and nicks between where the Arp8 module bind and the entry site of nucleosomes do not interfere with INO80-mediated nucleosome movement^17^.

Sensing linker DNA length is a critical property of INO80 as this remodeler is a major factor in establishing nucleosome free or depleted regions (NFR or NDR) at promoters^13,33^. Genomic studies indicate that Arp5 and Ies6 occupancies correlate with nucleosome positioning at transcription start sites and this positioning is changed when either these subunits or Ino80 are deleted^13^. INO80 is the only ATP-dependent chromatin remodeler that alone can properly reposition nucleosomes in yeast promoter regions when genomic DNA is reconstituted in vitro into chromatin^33^. Both yeast and human INO80 exhibit DNA sensing activity^22,32^ that likely helps establish promoter architecture. Our data along with recent studies from the Narlikar lab^23^ indicate that two seemingly distinct mechanisms involving the Nhp10 and Arp8 modules can be used by INO80 to slide nucleosomes in a linker DNA length dependent manner, while only the Arp8-Arp4-actin module is likely evolutionarily conserved. The Nhp10 and Arp8 modules appear to work by distinct mechanisms since the absence of the Nhp10 module causes INO80 to move nucleosomes regardless of extranucleosomal DNA length^23^ and the lack of Arp8 module binding to extranucleosomal DNA, from our data, arrests sliding even when there is sufficient extranucleosomal DNA. It is not clear how Nhp10 negatively regulates INO80 when linker DNA is shortened, but for the Arp8 module it appears that loss of Arp8 binding to linker DNA changes critical contacts of the Ino80 and Arp5 subunits with nucleosomes, thereby down-regulating chromatin remodeling by INO80. Our study suggests that actin-related proteins are used both to bind histones (Arp5) and DNA (Arp8) in order to regulate INO80 chromatin remodeling. For INO80, the Arp8 module is a DNA-binding sensor required for proper docking of the catalytic subunit and ATPase domain, which is quite distinct from how Arps regulate RSC and SWI/SNF through direct interaction with their ATPases domains^12,34–36^. Actin-related proteins in chromatin remodelers therefore can interact directly with the ATPase domain to fine tune its DNA translocation activity or make crucial interactions with nucleosomal substrates to allosterically regulate nucleosome mobilization.

## Methods

### Strain construction and enzyme purification

INO80 subunits Arp5, Arp4, and Nhp10 were tagged at their C-terminus using polymerase chain reaction (PCR)-mediated gene replacement in yeast. A 3HA peptide coding sequence and *URA3* selection marker were amplified from a pRS416-based plasmid (kind gift from Dr. Xuetong Shen). Purified PCR products were transformed into *Sacharomyces cerevisiae* BY4743, with the Ino80 subunit bearing a 2FLAG at the C-terminus^37^. Putative clones were selected by growth on uracil dropout media. Recombination junctions were verified by PCR and expression of HA-tagged proteins was confirmed by western blotting. Yeast expressing INO80-Arp8ΔN and a version of wild-type INO80, with 2FLAG-tag at the N-terminus of Arp8ΔN or full-length Arp8, respectively, were constructed by expressing 2FLAG-Arp8ΔN or 2FLAG-Arp8 from a pRS416-based centromeric plasmid containing the native *arp8* promoter, in *Sacharomyces cerevisiae* BY4733 with deletion of the endogenous *arp8* (INO80-ΔArp8, a kind gift from Dr. Xuetong Shen)^11^. Protein expression was confirmed by western blotting. Oligonucleotide primers used for epitope tagging and Arp8 N-terminal deletion are listed in Supplementary Table 1.

INO80 (with Ino80-2FLAG or 2FLAG-Arp8) complexes were purified by immune-affinity purification from yeast grown to O.D._600_ ~5–6 in 60 L YPD, harvested, and freeze-ground^17,37^. Briefly, immuno-affinity chromatography was performed using ANTI-FLAG M2 Affinity Gel (Sigma) and the complexes were eluted with 1 mg ml^−1^ FLAG peptide. The purity and integrity of the complexes were determined by loading denatured samples on 4–20% SDS-polyacrylamide gels and staining with Coomassie Blue. The activities of the purified complexes were determined by EMSAs to test for nucleosome binding and remodeling, and thin-layer chromatography (TLC) for ATP hydrolysis (see below).

### Nucleosome reconstitution

70N0 nucleosomes (N = nucleosome core particle with 147 bp of 601-nucleosome positioning sequence DNA, and number on either side indicate length(s) of extranucleosomal DNA) were assembled at 37 °C by salt-dilution with 7–10 µg of recombinant *Xenopus laevis* histone octamers (wild type or cysteine mutant octamer, see below), 100–200 fmol of 5′[^32^P]-labeled 217 bp 601-DNA (or 601-DNA containing site-specific photo-reactive labels, see below), 5–10 µg of sheared salmon sperm DNA (for heterogeneous nucleosomes) or 601-DNA without [^32^P]-radiolabel as carrier (for homogeneous nucleosomes used in ATPase assay and nucleosome binding assays, see below). PCR oligos are listed in Supplementary Table 1. DNA and histone octamer were mixed at ~1:1 w/w ratio in 2 M NaCl. Nucleosomes were dialyzed in steps to reach a final concentration of 280 mM NaCl in 25 mM Tris-HCl (pH 8). Samples were examined on 4% native polyacrylamide gels where nucleosome and free DNA show different electrophoretic mobility, and nucleosome assembly was confirmed by phosphorimaging.

### Site-specific DNA photoaffinity crosslinking

Site-specific photo-reactive DNA probes with 601-nucleosome positioning sequence for 70N0 nucleosomes were synthesized by enzymatic incorporation of modified nucleotides into double stranded DNA^17,18^. dUMP and dCMP analogs coupled with *p*-azidophenacyl bromide were incorporated along with [α-^32^P] dGTP/dATP, in tandem, at specific positions. Photo-reactive DNA was reconstituted into nucleosomes and bound with saturating amounts of wild type or mutant INO80 at 30 °C for 30 min. The extent of enzyme binding was assessed on 4% native polyacrylamide gels. In all experiments >90% of nucleosomes were bound by the enzyme. After binding, INO80 was crosslinked to DNA by UV irradiation (3 min at 310 nm, 2.65 mW cm^−2^), and DNA was digested with DNaseI and S1 nuclease for transfer of the radioactive label to the crosslinked protein(s)^18^. Protein subunits were separated on 4–20% SDS-polyacrylamide gels and radiolabeled subunit(s) were visualized by phosphorimaging. Data images in figures are representative of ≥3 experiments.

Binding reactions were scaled up for immunoprecipitation of photoaffinity-labeled subunits. After DNase I and S1 nuclease digestion, pH of the reactions was adjusted to 7.0 with 0.5 M Tris-base and reactions were diluted 4-fold with pre-cooled IP buffer containing 25 mM Na-HEPES (pH 7.6), 300 mM NaCl, 10% glycerol, 0.1% NP-40, 10 mM 2-mercaptoethanol (BME), 1 μg ml^−1^ leupeptin, 1 μg ml^−1^ pepstatin A, and 1 mM PMSF. One microlitre and three microlitres of anti-Arp4 antibody (generously provided by Dr. Bruce Stillman) were added to reactions, which were incubated at 4 °C overnight, followed by an additional hour at 4 °C with equilibrated EZView Protein A-agarose (Sigma) with gentle agitation. Agarose beads were washed three times with IP buffer and resuspended in SDS sample buffer. Samples were analyzed on 4–20% gradient SDS-polyacrylamide gels.

### Isolation of Arp8 and Arp4 subunits for peptide mapping

INO80 photoaffinity-labeling reactions were scaled up for peptide mapping. Following digestion of DNA with DNaseI and S1 nuclease, and label transfer, photoaffinity-labeled INO80 complex was denatured with 0.4% SDS and sample volumes were reduced to 30 μl in a Centrivap concentrator (Labconco). Samples were heated at 90 °C for 3 minutes in SDS sample buffer and loaded on preparative 6% SDS-PAGE gels. At each probe position, photoaffinity-labeled Arp8 and Arp4 were visualized immediately after electrophoresis by phosphorimaging. The protein bands were excised and electro-eluted (Bio-Rad 422 Electro Eluter) in a volatile elution buffer containing 50 mM NH_4_HCO_3_ and 1% SDS. Eluted samples were dried in a Centrivap concentrator (Labconco) and stored at −20 °C until proteolysis of samples.

### Peptide mapping with cyanogen bromide

Dried, photoaffinity-labeled Arp8 and Arp4 samples were resuspended in 2% SDS and 0.2 mM 2-mercaptoethanol, and divided into several 30 μl aliquots. For each sample, 2 μl of 1 M HCl and 2 μl of various concentrations (20 mM to 2 M) of freshly prepared cyanogen bromide (CNBr) solution (in acetonitrile) was mixed with the protein samples and incubated at 25 °C in the dark for up to 2 h to vary the degree of digestion. CNBr reactions were stopped by diluting the samples with 170 μl of water and drying in a Centrivap concentrator (Labconco). The dried CNBr cleavage samples were resuspended in SDS sample buffer and neutralized by adding 0.5 M Tris-base. Samples were analyzed by 4–20% gradient SDS-PAGE for Arp8 and 10–20% gradient SDS-PAGE for Arp4. The sizes of the [^32^P]-labeled fragments were compared to [^35^S]-labeled, in vitro synthesized peptide markers corresponding to the expected protein fragments. Data images in figures are representative of three experiments.

### Peptide mapping with 2-Nitro-5-thiocyanatobenzoic acid

Dried, photoaffinity-labeled Arp8 and Arp4 samples were resuspended in freshly prepared denaturing buffer containing 8 M urea, 10 mM 2-mercaptoethanol, and 0.2 M Tris-acetate (pH 8.5) by incubating at 37 °C for 3 hours prior to dividing into several 36 μl aliquots. A volume of 2 μl of various concentrations (30 mM to 1 M) of NTCB (dissolved in ethanol) was added and incubated at room temperature for 15 minutes. At this stage, NTCB modifies cysteine without cleavage. To initiate cleavage, the pH of the solution was adjusted to ~9.0–9.5 with 1 M NaOH as measured by a micro pH-probe, followed by incubation at 37 °C for 4 hours. The cleavage reactions were stopped by adding SDS sample buffer and heating to 90 °C. Samples were analyzed on 4–20% gradient SDS-PAGE gels for Arp8 and 10–20% gradient gels for Arp4. The sizes of the [^32^P]-labeled fragments were compared to [^35^S]-labeled, in vitro synthesized peptide markers corresponding to the expected protein fragments. Data images in figures are representative of three experiments.

### Peptide mapping of Ino80 subunit with ArgC protease

Photoaffinity-labeled INO80 complex (after digestion of DNA and label transfer) was denatured with 0.4% SDS and heating at 90 °C for 3 minutes, followed by buffer exchange using Amicon Ultra filters to remove SDS and FLAG peptides. C-terminal FLAG-tagged Ino80 was purified by immobilization on ANTI-FLAG^®^ M2 Affinity Gel (Sigma). Protein-bound beads were washed and resuspended in ArgC incubation buffer containing 50 mM Tris-HCl (pH 7.8), 5 mM CaCl_2_ and 2 mM EDTA. Protein cleavage was initiated by the addition 5 mM DTT (final concentration) and varying concentrations of ArgC protease (Promega, sequencing grade) with incubation at 37 °C for 2 hours. Reactions were stopped by the addition of 1 mM PMSF and 10 mM EDTA. Immobilized C-terminal fragments were separated from the released fragments and washed three times in the same buffer as the digestion. The bead fractions were resuspended in SDS sample buffer, resolved on 4–20% Tris-glycine SDS-polyacrylamide gels, and analyzed by both phosphorimaging, as well as transfer and anti-FLAG immunoblotting (see below). Apparent molecular masses of the Ino80-FLAG fragments were estimated by comparing their migration relative to the [^35^S]-labeled Ino80-FLAG markers of known molecular weights prepared by in vitro coupled transcription and translation using TnT^®^ T7 Quick Coupled Transcription/Translation System (Promega). Data images in figures are representative of ≥3 experiments.

### Western blots

Protease (ArgC) digested Ino80 fragments were resolved in 20 cm × 20 cm 4–20% Tris-glycine SDS-polyacrylamide gels and transferred onto PVDF membranes using Bio-Rad Trans-Blot® electrophoretic transfer cell for 3 hours at 4 °C, using a transfer buffer containing 25 mM Tris, 192 mM glycine, 20% methanol, 0.1% SDS (pH 8.3) at 50 V constant voltage. The membranes were blocked with 5% fat-free milk in TBST (20 mM Tris-HCl pH 7.5, 150 mM NaCl, 0.1% Tween-20), overnight at 4 °C, washed with TBST, and incubated with mouse monoclonal ANTI-FLAG® M2-Peroxidase (HRP) antibody (Sigma Aldrich catalog # A8592) diluted 1:1000 for 1 hour at room temperature. The blots were washed with TBST, and developed with SuperSignal™ West Femto Maximum Sensitivity Substrate (Thermo Fisher) before visualization using an Image Quant LAS 4000 (GE healthcare Life Sciences). Uncropped images of western blots are shown in Supplementary Fig. 6.

### Synthesis of Arp8 and Arp4 peptide-mapping markers

The *arp8* and *arp4* genes, plus 100 bp of DNA flanking each end of the coding sequences, were PCR-amplified from genomic DNA isolated from the same yeast strain used for INO80-FLAG purification. This DNA was used as templates for PCR amplification of expression cassettes with coding sequences corresponding to Arp8 and Arp4 fragments expected from NTCB or CNBr cleavage, under the control of T7 promoter and terminator. Arp8 and Arp4 peptides of specific lengths and molecular weights were synthesized by coupled in vitro transcription/translation using the TnT^®^ Quick Coupled Transcription/Translation System (Promega) with [^35^S]-Methionine (Perkin Elmer). Transcription/translation reactions were carried out at 30 °C for 90 min, followed by the addition of 20 μg ml^−1^ leupeptin. Samples were analyzed on 4–20% Tris-glycine gradient SDS-polyacrylamide gels followed by phosphorimaging. Peptide standards corresponding to both incomplete or limited digestion as well as complete digestion conditions were prepared. Oligonucleotide primers for Arp8 and Arp4 peptide synthesis are listed in Supplementary Table 1.

### Synthesis of Ino80-FLAG peptide-mapping markers

The *ino80* gene with 2FLAG coding sequence at the 3′ end, plus 100 bp of DNA flanking each end of the coding sequence, was PCR-amplified from genomic DNA isolated from the same yeast strain used for INO80-FLAG purification. This DNA was used as template for PCR amplification of expression cassettes with coding sequences corresponding to truncated Ino80-FLAG fragments of varying lengths from the C-terminus. Peptides were synthesized by coupled in vitro transcription/translation as described above. Peptide standards of all sizes were pooled and run alongside Ino80 peptide-mapping reactions. Relative electrophoretic mobilities (*R*_f_) of the peptide marker from three technical replicates were calculated and plotted against log_10_ of their molecular weights to generate a standard curve^17^. Molecular weights and sizes of protein fragments generated by ArgC cleavage were estimated from the standard curve based on their *R*_f._ Protease cleavage sites were mapped to arginine residues closest to the estimated cleavage sites. Oligonucleotide primers for Ino80 peptide synthesis are listed in Supplementary Table 1.

### Yeast growth assays

Purified single colonies of wild-type INO80, INO80-ΔArp8, INO80-ΔArp8(pArp8), INO80-Arp8ΔN, INO80-ΔSpacer, and INO80-ΔTELY strains were diluted in water to O.D._600_ = 0.8 and 10-fold serial dilutions were spotted onto plates containing YPD, synthetic media lacking inositol, and YPD with 0.05% MMS or 0.1 M HU. Images of the plates were taken after 3–4 days at 30 °C. Experiments were repeated three times.

### Kinetics of nucleosome remodeling using gel-shift assays

INO80 complexes (26 nM) were pre-bound to 70N0 nucleosomes (25 nM) at 30 °C for 30 min. ATP was added to a final concentration of 80 μM and incubated at 30 °C for remodeling. Samples were taken at the indicated time periods (Supplementary Fig. 3a). Remodeling reactions were stopped by adding ATP-γ-S and sonicated salmon sperm DNA (stop mix) to final concentrations of 1.5 mM and 300 ng µl^−1^, respectively. Samples were analyzed on 5% native polyacrylamide gels in 0.5X TBE buffer. Data images in figures are representative of three experiments.

### Nucleosome-stimulated ATP hydrolysis (ATPase assay)

ATPase assays were carried out with homogeneous 70N0 nucleosomes (discussed above), but under the same conditions as for the remodeling assays. After pre-binding of the enzymes with nucleosomes at 30 °C for 30 min, 80 μM ATP was added spiked with [γ-^32^P]-labeled ATP. Reactions were stopped by adding SDS and EDTA to final concentrations of 2% and 100 mM, at indicated time points which were identical to the remodeling time course experiment (Fig. 2d and Supplementary Fig. 3a). The extent of [γ-^32^P]-ATP hydrolysis was determined by quantifying the amount of inorganic ^32^P (Pi) released, which is resolved from un-hydrolyzed ATP and ADP by TLC on polyethyleneimine cellulose plate (J.T. Baker, Germany) developed with 0.5 M LiCl and 0.5 M formic acid. Experiments were repeated four times.

### High-resolution mapping of changes in H2B53–DNA contacts

Histone octamers containing cysteine at residue 53 of H2B were conjugated to p-azido phenacyl bromide (APB) immediately after octamer refolding. Wild type or mutant INO80 was incubated with nucleosomes at 30 °C for 15 min in conditions so that nucleosomes would be completely bound. Nucleosome movement was initiated by adding 80 μM ATP and was stopped with 1.5 mM ATP-γ-S and excess EDTA at the indicated time points (Supplementary Fig. 3b). Aliquots were analyzed on 5% native polyacrylamide gels (as in Supplementary Fig. 3a). For site-directed histone-DNA crosslinking^26^, samples were irradiated with UV (3 min at 310 nm, 2.65 mW cm^−2^). Samples were denatured with 0.1% SDS at 37 °C for 20 min in 30 mM NaCl and 20 mM Tris-HCl (pH 8.0). Crosslinked protein-DNA were enriched and separated from un-crosslinked DNA by phenol-chroloform (4:1) extraction. The aqueous phase containing un-crosslinked DNA was discarded. Crosslinked DNA was ethanol precipitated with 1 M LiCl in the presence of sheared salmon sperm DNA as carrier. Crosslinked DNA was cleaved with 1 M pyrrolidine (Sigma) at 90 °C for 20 min. DNA samples were analyzed alongside a sequence ladder made from the same DNA, on denaturing 6.5% polyacrylamide gels containing 8 M urea. Gels were visualized by phosphorimaging and quantified using ImageQuant software (Version 5.2). Total lane intensity was normalized to correct for loading bias using Microsoft Excel. Data images in figures are representative of three experiments.

### Nucleosome binding assays

Increasing concentrations of wild type or mutant INO80 as indicated (Supplementary Fig. 4a) were incubated with 40 nM homogeneous 70N0 nucleosomes (discussed above) for 30 min at 30 °C in 10 mM Na-HEPES (pH7.8), 4 mM MgCl_2_, 60 mM NaCl, 0.2 mM EGTA, 0.04 mM EDTA, 8% glycerol, and 0.1 μg μl^−1^ bovine serum albumin. Reactions were analyzed by resolving enzyme-bound nucleosomes from free nucleosomes on 4% native polyacrylamide gels in 1× Tris-EDTA buffer. Data images in figures are representative of three experiments.

### Histone crosslinking

Recombinant *Xenopus laevis* histones with amino acids replaced by cysteine at specific positions (Fig. 4a) were expressed, purified, and reconstituted into octamers with other histones^27^ as discussed above. Reducing conditions with 5 mM 2-mercaptoethanol were maintained for cysteine mutant histones and octamers. Refolded octamers were immediately mixed with a 20-fold molar excess of PEAS [N-((2-pyridyldithio)ethyl)-4-azidosalicylamide] (Thermo Fisher) and incubated at room temperature for 30 minutes. Excess unconjugated PEAS was removed by buffer exchange (100-fold) with octamer dilution buffer (5 M Tris-HCl pH 8, 0.5 mM EDTA, 1 M NaCl) and subsequent concentration using a 30 KDa cutoff Amicon ultrafilter column. The octamers were stored at −20 °C in 50% glycerol. Homogeneous 70N0 nucleosomes formed with the 601-nucleosome positioning sequence containing Cy5 label at one end were reconstituted as discussed above. [^125^I] (Perkin Elmer) was added into IODOGEN tubes (Pierce) in a 2.5 times molar excess over that of the nucelosomes to be iodinated, and pre-equilibrated with an equal volume of 100 mM sodium phosphate (pH 7.4). After 6 minutes of incubation, nucleosomes were added and incubated at room temperature for 5 minutes. The iodination reaction was stopped by adding 1μl of 2.5 mM tyrosine and 1μl of 80 mM methionine. Unconjugated ^125^I was removed using Sephadex G25 spin-columns centrifuged at 800 × *g* for 2 minutes at room temperature. The amount of nucleosome-conjugated PEAS was determined by measuring the specific activity of ^125^I in nucleosomes compared to the specific activities of known molar quantities of unconjugated PEAS-[^125^ I]. Nucleosomes (70N0) with photo-reactive octamers were bound to saturating amounts of wild type or mutant INO80 at 30 °C and assessed as before. Samples were irradiated directly under UV (3 minutes at 310 nm, 2.65 mW cm^-2^) for crosslinking. After crosslinking, the samples were treated with 100 mM DTT and incubated at 37 °C for 30 minutes to transfer [^125^I]-radiolabel to the crosslinked proteins. The samples were resolved on 4–20% SDS-polyacrylamide gels, dried, and viewed by phosphorimaging following 14–16 hours of exposure to identify the radiolabeled subunits. Data images in figures are representative of three experiments.

### Statistical analysis

For each independent in vitro experiment, at least three technical replicates were performed as mentioned in the figure legends and/or experimental procedures. DNA crosslinking experiments were repeated with two independent preparations of the INO80 complex, which showed identical results. Peptide-mapping markers of known molecular weights were prepared two times and relative electrophoretic migration (R_f_ values) of markers from each set were compared to ensure consistency of results. Growth assays were repeated three times, each from separate single colonies of yeast with wild type or mutant INO80.

### Data availability

The data sets generated and/or analyzed during the current study are available from the corresponding author on reasonable request.

## Electronic supplementary material

Supplementary Information
