# Supplementary material for: The Arp8 and Arp4 module acts as a DNA sensor controlling INO80 chromatin remodeling
Source: Nat Commun. 2018 Aug 17;9:3309. doi: 10.1038/s41467-018-05710-7 (PMC6098158; doi:10.1038/s41467-018-05710-7)
Supplement: Supplementary file 1 — Supplementary Information [file 41467_2018_5710_MOESM1_ESM.pdf]

Supplementary Information

The Arp8 and Arp4 module acts as a DNA sensor controlling INO80 chromatin remodeling

Brahma et.al.

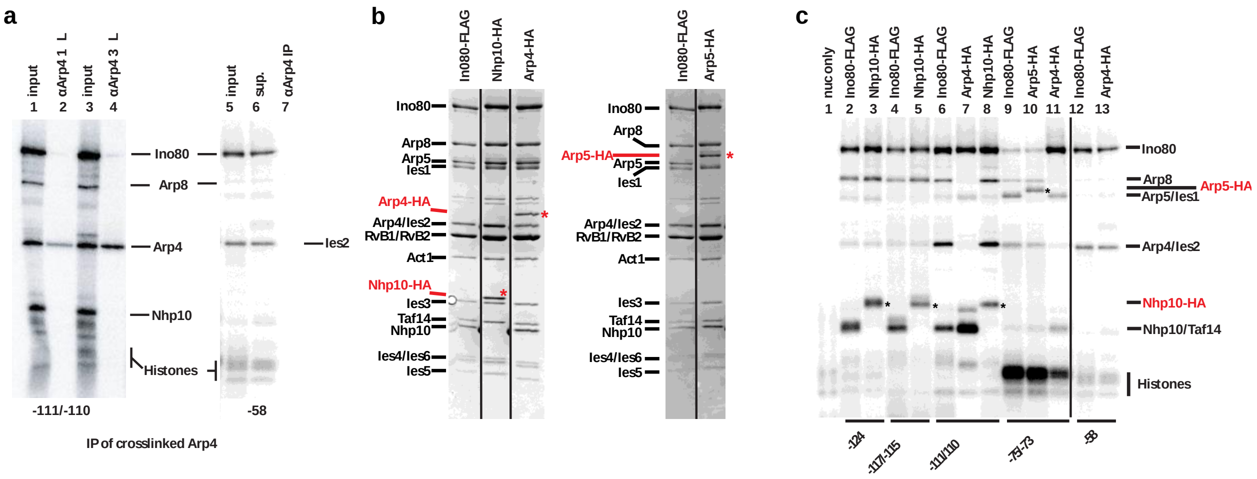

Supplementary Figure 1. Identification of INO80 subunits that crosslink with DNA.

(**a**) In order to distinguish crosslinked Arp4 from Ies2, Arp4 was immunoprecipitated (IP) from the crosslinked samples. The INO80 bound to nucleosomes was crosslinked to DNA at nt -111/-110 (lanes 1-4) and nt-58 (compare lanes 5-7) and immunoprecipitated with anti-Arp4. The precipitated samples (lanes 2, 4, and 7) were compared to either the initial input (lanes 1, 3, and 5) or the supernatant (lane 6). (**b**) The C-terminus of Ino80 was tagged with two copies of the FLAG epitope and the complex was purified by immunoaffinity chromatography. The Nhp10, Arp4, and Arp5 subunits are also tagged with three copies of the HA tag in some instances (*). (c) The Nhp10 and Arp5 subunits were distinguished from Taf14 and Ies1 with three copies of the HA epitope. HA tagging Arp4 to determine its identity when crosslinked to DNA is less straightforward as C-terminal HA tags on Arp4 disrupt the binding of Arp4 and Arp8 to extranucleosomal DNA (lanes 7 and 11), while enhancing the interactions Nhp10 (lane 7) and Ino80 (lane 11) with DNA.

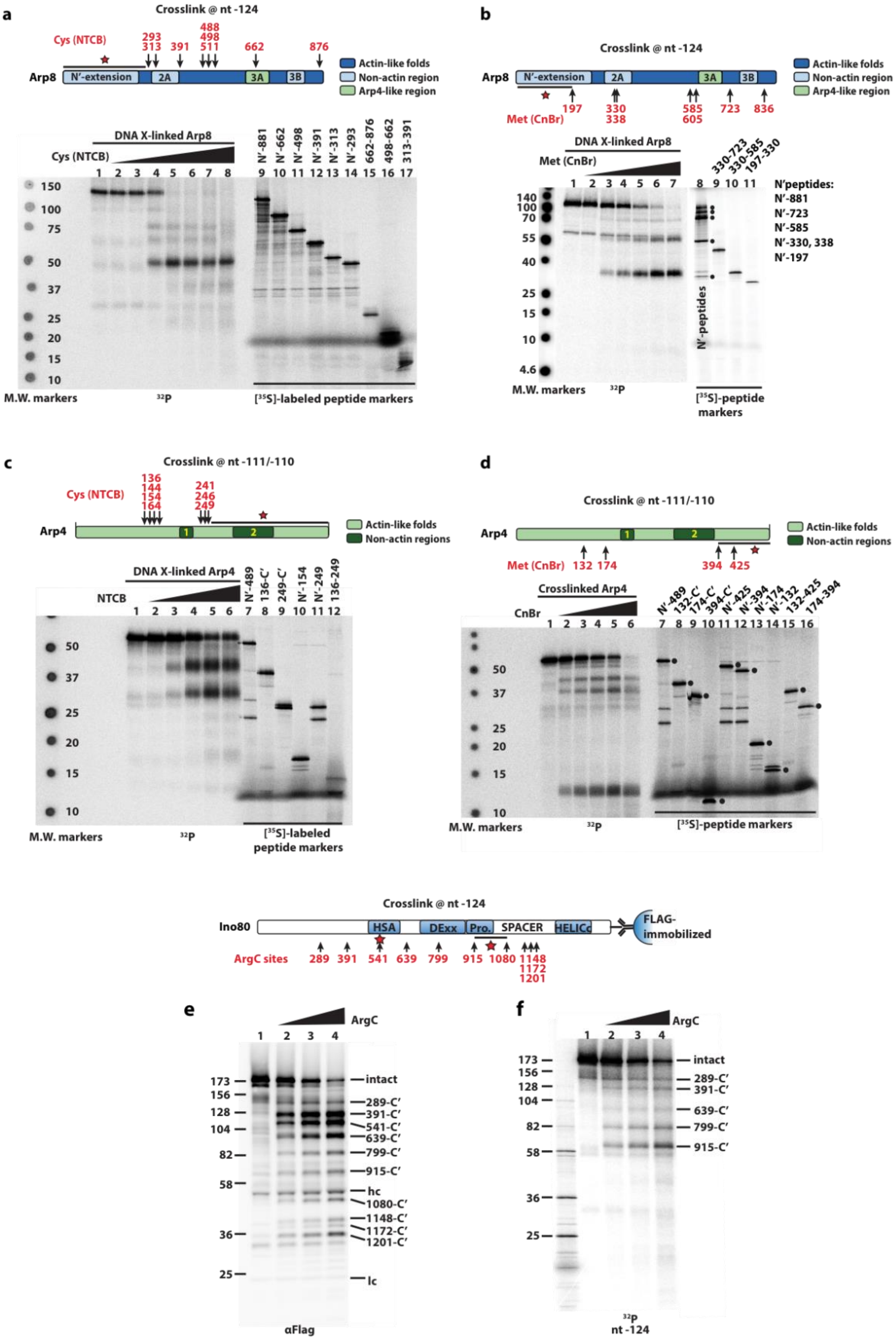

a
Crosslink @ nt -124
Cys (NTCB)
293 313 391 488 498 511 662 876
Arp8
N'-extension
2A
3A
3B
Actin-like folds
Non-actin region
Arp4-like region
DNA X-linked Arp8
Cys (NTCB)
N'-881 N'-662 N'-498 N'-391 N'-313 N'-293 662-876 498-662 313-391
1 2 3 4 5 6 7 8 9 10 11 12 13 14 15 16 17
150
100
75
50
37
25
20
15
10
M.W. markers
^{32}P
[35S]-labeled peptide markers
b
Crosslink @ nt -124
Arp8
N'-extension
2A
3A
3B
Actin-like folds
Non-actin region
Arp4-like region
197 330 338 585 605 723 836
Met (CnBr)
DNA X-linked Arp8
Met (CnBr)
330-723 330-585 197-330
1 2 3 4 5 6 7 8 9 10 11
N'peptides:
N'-881
N'-723
N'-585
N'-330, 338
N'-197
140
100
70
55
40
25
15
10
4.6
N'-peptides
M.W. markers
^{32}P
[35S]-peptide markers
c
Crosslink @ nt -111/-110
Cys (NTCB)
136 144 154 164
241 246 249
Arp4
1
2
Actin-like folds
Non-actin regions
DNA X-linked Arp4
NTCB
N'-489 136-C' 249-C' N'-154 N'-249 136-249
1 2 3 4 5 6 7 8 9 10 11 12
50
37
25
20
15
10
M.W. markers
^{32}P
[35S]-labeled peptide markers
d
Crosslink @ nt -111/-110
Arp4
1
2
Actin-like folds
Non-actin regions
Met (CnBr) 132 174 394 425
Crosslinked Arp4
CnBr
N'-489 132-C' 174-C' 394-C' N'-425 N'-394 N'-174 N'-132 132-425 174-394
1 2 3 4 5 6 7 8 9 10 11 12 13 14 15 16
50
37
25
20
15
10
M.W. markers
^{32}P
[35S]-peptide markers
Crosslink @ nt -124
Ino80
HSA
DExx
Pro.
SPACER
HELICc
FLAG-immobilized
ArgC sites 289 391 541 639 799 915 1080 1148 1172 1201
e
ArgC
1 2 3 4
173
156
128
104
82
58
36
25
intact
289-C'
391-C'
541-C'
639-C'
799-C'
915-C'
hc
1080-C'
1148-C'
1172-C'
1201-C'
lc
αFlag
f
ArgC
1 2 3 4
173
156
128
104
82
58
36
25
intact
289-C'
391-C'
639-C'
799-C'
915-C'
^{32}P
nt -124

Supplementary Figure 2. Mapping the regions of Arp8, Arp4 and Ino80 that are bound to extranucleosomal DNA.

(**a-d**) Uncropped images of gels in **Fig. 1d** (a, b) and **Fig. 1e** (c, d). The [^{35}S] labeled custom-synthesized peptide molecular weight markers used to identify the products from proteolytic cleavages of crosslinked Arp8 and Arp4 at Cys and Met residues are shown here alongside the Arp8 and Arp4 fragments, as well as general molecular weight markers (their positions were marked with radioactive ink and their molecular weights are expressed in kDa). (**e, f**) The region of Ino80 crosslinked to nt -124 was mapped using ArgC protease, similar to **Fig. 1f**. Schematics show the locations of Arg residues that were cleaved, the HSA and ATPase domains, and the two regions that crosslink to DNA (highlighted by a black bar and red asterisks). Numbers on the left correspond to custom-synthesized markers (their molecular weights expressed in kDa, and on the right, indicate the lengths of the peptides resulting from ArgC cleavage, in number of amino acids from the C-terminus. All FLAG-tagged fragments resulting from cleavage are identified by immunoblotting (αFLAG, left) and compared to photo-crosslinked fragments (^{32}P, right) to discern the region of crosslinking (see **Fig. 1c, f**).

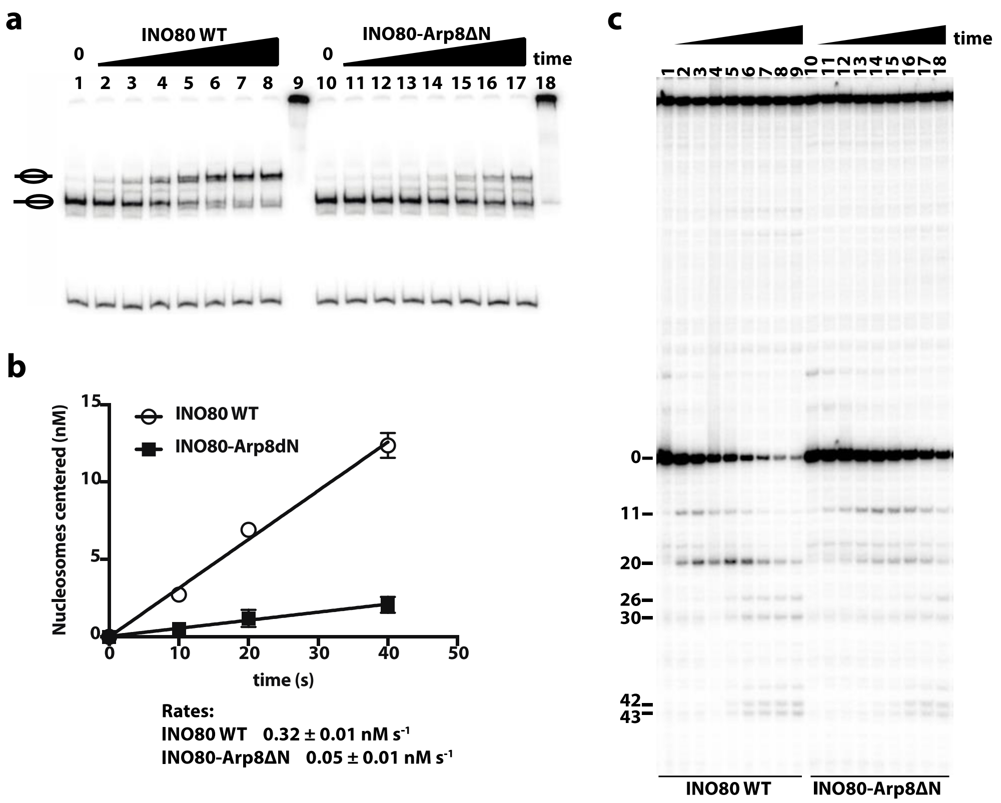

Supplementary Figure 3. INO80 requires the N-terminus of Arp8 to displace nucleosomal DNA from the H2A-H2B interface.

(a) Nucleosome mobilization by wild type (lanes 1–8) and Arp8ΔN INO80 (lanes 10–17) were assessed by native electrophoretic mobility shift (EMSA) assays using end-positioned (70N0) nucleosomes saturated with wild type or Arp8ΔN INO80 (lanes 9 and 18), and remodeled with 80 μM ATP at 30°C for 0, 10, 20, 40, 80, 160, 320, and 640s. Data from three technical replicates of (a) were used to determine rates of remodeling as in **Fig. 2c**, and panel (b) below. **(b)** Rates of nucleosome movement were determined by measuring the slope of line at the earlier time points (0-40 sec). Error bars denote s.e.m from 3 experiments. (**c**) Nucleosomes modified at amino acid residue 53 of H2B were used to examine DNA movement on the octamer with wild type and mutant INO80, and as shown (top panel), are on the side of nucleosomes with the 70 bp

of extranucleosomal DNA. Arrows indicate the direction of DNA movement and location of DNA crosslink with H2B53. Dotted sphere shows the altered nucleosome position upon remodeling. Nucleosomes were saturated with wild-type INO80 (lanes 1-9) or Arp8ΔN INO80 (lanes 10-18), and remodeled with 80 μM ATP at 30 °C for 0, 10, 20, 40, 80, 160, 320, 640, and 1280s. Un-remodeled nucleosomes are labeled 0. DNA in these nucleosomes is cleaved 54 bps from the dyad axis. Shorter DNA products result from DNA moving into nucleosomes and across residue 53 of histone H2B. Numbers beside the bands correspond to the number of nucleotides moved from the starting position. Data from three technical replicates of (c) were used to plot the graphs in **Fig. 2 e-h**.

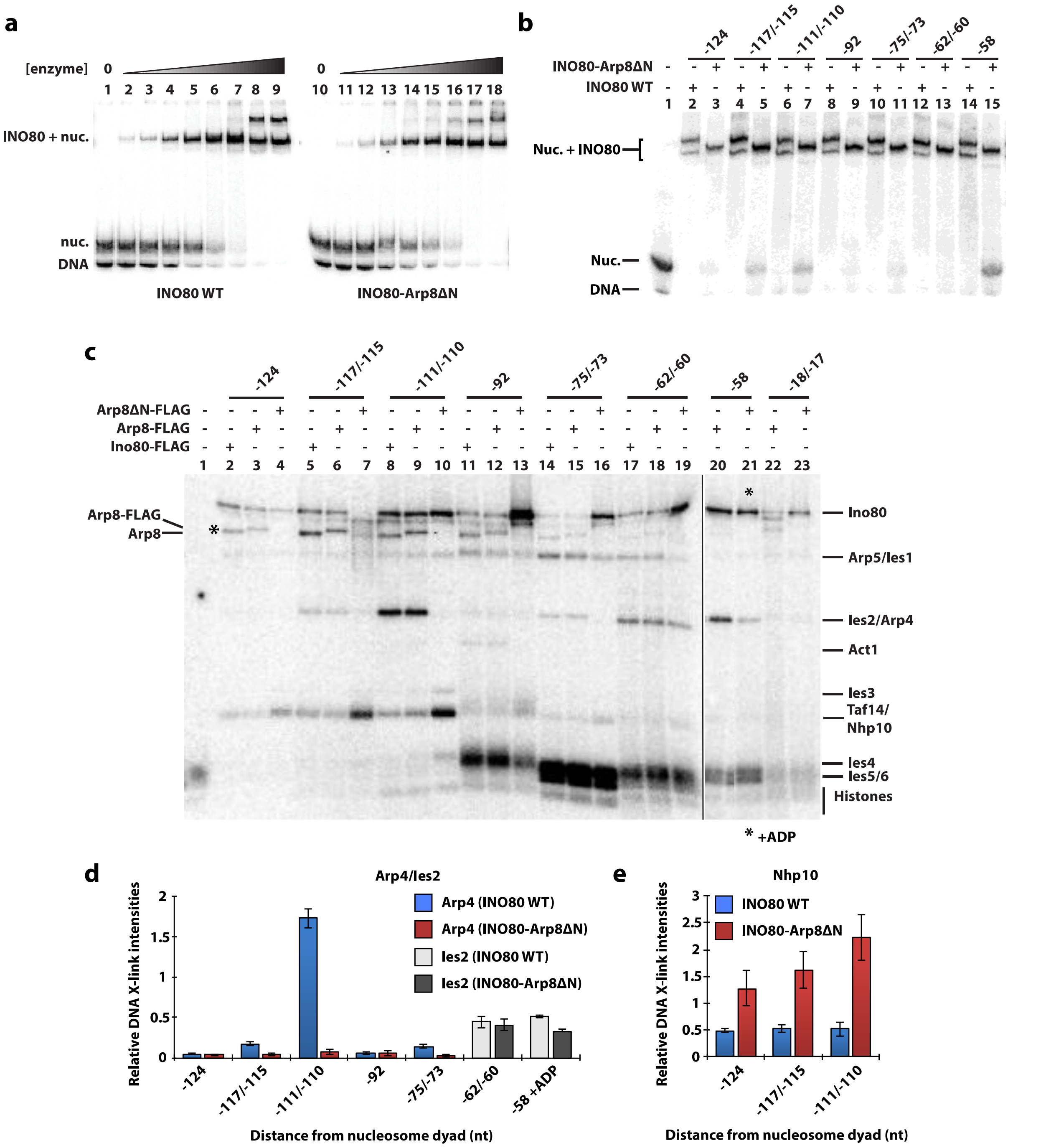

Supplementary Figure 4. The N-terminus of Arp8 is required for the stable binding of Arp4, but not Nhp10, to extranucleosomal DNA.

(a) The affinity of wild type INO80 (lanes 1–9) and Arp8ΔN INO80 (lanes 10–18) for homogeneous 70N0 nucleosomes was measured by native EMSA. INO80 (0, 1.25, 2.5, 5, 10, 20,

40, 80, and 160 nM) was incubated with nucleosomes (40 nM) for 30 min at 30°C. Data from three technical replicates of (a) were used to generate the plot in **Fig. 3a**. (**b**) Heterogeneous nucleosomes assembled with DNA containing photo-reactive nucleotides at specific positions were saturated with wild type INO80 or Arp8ΔN INO80 (even versus odd lanes) for nucleosomal DNA crosslinking experiments (see **Fig. 3b**). (**c**) Site-specific interactions of INO80 and Arp8ΔN INO80 with extranucleosomal and nucleosomal DNA were compared as in **Fig. 3b**. Wild type INO80 complexes with 2FLAG-tag either at the C-terminal of Ino80 (Ino80-FLAG) or at the N-terminus of full-length Arp8 (Arp8-FLAG) were compared to confirm that Arp8 is crosslinked (lanes 2-19). Arp8 showed shift in electrophoretic mobility due to the 2FLAG-tag. (**d, e**) Crosslinking efficiencies of Arp4 and Ies2 (d) or Nhp10 (e) in wild-type and Arp8ΔN INO80 at the indicated DNA positions are plotted from the data shown in (c) and **Fig. 3b**. Values are mean of 3 experiments, normalized to the Ino80 subunit of wild type at nt -58 without ADP (lane 14 in **Fig.3b**). Error bars denote ± s.d.

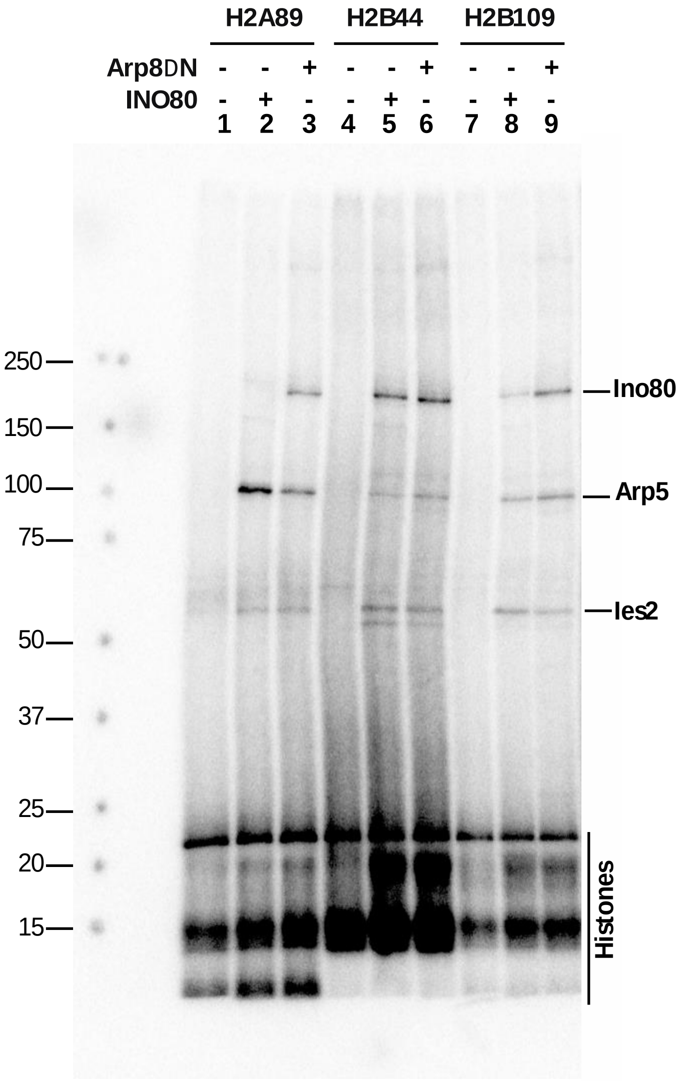

Supplementary Figure 5. Arp5 binding near residue 89 of histone H2A in the acidic-pocket region requires the N-terminus of Arp8.

Shown here is a representative uncropped image of the wild-type INO80 and Arp8ΔN INO80 crosslinked with histones (as in **Fig. 4d**), along with the positions of molecular weight markers (marked with radioactive ink, and their molecular weights expressed in kDa). The Ino80, Arp5 and Ies2 subunits of INO80 crosslinked mainly at the three positions probed on the H2A-H2B dimer surface.

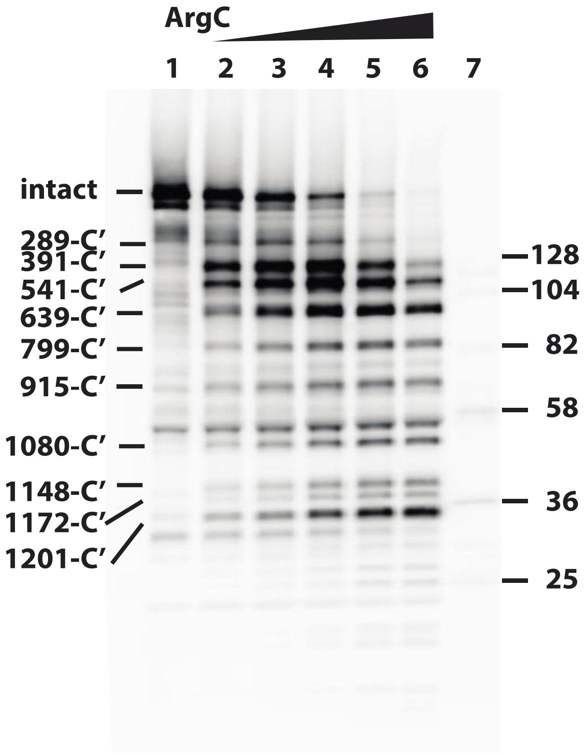

Supplementary Figure 6. Uncropped Western-blot image of Ino80 peptide mapping experiment shown in **Fig. 1f** (left panel). Also shown here are the FLAG-tagged custom-synthesized molecular weight markers (lane 7) and their molecular weights in kDa (numbers on the right). Numbers on the left correspond to peptides resulting from ArgC cleavage, and their lengths in number of amino acids from the C-terminus are indicated.

Supplementary Table 1.

List of oligonucleotide primers

HA-tagging of INO80 subunits	
Name	Sequence (5 ′ - 3 ′)
ARP5 FW	ATC AAA GAG CAT AAG TTA GGG AAT ACG AAG TAT TTT GAA GAC GAA TTC CAT ATG GCG GCC GCA
ARP5 RV	TTC TCT TTT TTT GTT TTT AAG GCG TTT CAG TTT GCT GTC TCC CTG TGC GGT ATT TCA CAC CG
ARP4 FW	GAA GAG GTG GGC GTC GAA AGA TTG CTT AAC GAT AGG TTT AGA GAA TTC CAT ATG GCG GCC GCA
ARP4 RV	AAC TGA AAG GCG ACT TGT CAT TCA ACA ACG TTT TCT ATT C CTG TGC GGT ATT TCA CAC CG
NHP10 FW	GAT TCT AAA GGA GGT GAA GAT GGA AGT TTA GTT TCC TCT AAC GAA TTC CAT ATG GCG GCC GCA
NHP10 RV	TAT CTT CAA AGA AAA TAG AAA AAA ATG GAA TTT TTA ATT T CTG TGC GGT ATT TCA CAC CG
TAF14 FW	TTA TTG AAA AGT CTA TGG GAC TAC GTT AAG AAA AAT ACC GAG GAA TTC CAT ATG GCG GCC GCA
TAF14 RV	CAA ACA TAA AAG CGC GCA TTT AAC GCC CTT TTA CCT TTT A CTG TGC GGT ATT TCA CAC CG
Arp8 N-terminus deletion	
Arp8 del 8-197 FW	TTA ATC AAC CAA AAA AAC TAC TC
Arp8 del 8-197 RV	TTC TGC TTC TTC TTG CGA
Arp8 peptide markers for CnBr cleavage	
Name	Sequence (5 ′ - 3 ′)
Arp8 N-FLAG FW	TCGATGCATACTAATACGACTCACTATAGGGAGCCGCCACCATG GAC TAC AAG GAC GAC G
N-197 RV	TTTTTTTTTTTTTTTTTTTTTTTTTTTTTTTCA CAT TAA AGG TTG GAA CAG GCC
N-330 RV	TTTTTTTTTTTTTTTTTTTTTTTTTTTTTTTCA CAT CTC CGA CTT TAT GTT G
N-338 RV	TTTTTTTTTTTTTTTTTTTTTTTTTTTTTTTCA CAT ACG TTC TCT AAA ATT C
N-585 RV	TTTTTTTTTTTTTTTTTTTTTTTTTTTTTTTCA CAT AAA ATT GTA CAA CTG AAC G
N-605 RV	TTTTTTTTTTTTTTTTTTTTTTTTTTTTTTTCA CAT AAC TTC GTC GAA CAG C
N-732 RV	TTTTTTTTTTTTTTTTTTTTTTTTTTTTTTTCA CAT TCT TGT TAC ATC AGC
N-836 RV	TTTTTTTTTTTTTTTTTTTTTTTTTTTTTTTCA CAT ATC ACG TGG TGG TGG G
Arp8 338-585 FW	TCGATGCATACTAATACGACTCACTATAGGGAGCCGCCACCATG AGG TAT TAT AAA AGA A

Arp8 338-585 FW	TTTTTTTTTTTTTTTTTTTTTTTTTTTTTTCA CAT AAA ATT GTA CAA CTG AAC
Arp8 330-605 FW	TCGATGCATACTAATACGACTCACTATAGGGAGCCGCCACCATG GAA AAG AAT TTT AGA G
Arp8 330-605 RV	TTTTTTTTTTTTTTTTTTTTTTTTTTTTTTCA CAT AAC TTC GTC GAA CAG C
Arp8 338-723 FW	TCGATGCATACTAATACGACTCACTATAGGGAGCCGCCACCATG AGG TAT TAT AAA AGA A
Arp8 338-723 RV	TTTTTTTTTTTTTTTTTTTTTTTTTTTTTTCA CAT TCT TGT TAC ATC AGC TG
Arp8 peptide markers for NTCB cleavage	
Name	Sequence (5 ′ - 3 ′)
Arp8 N-FLAG FW	TCGATGCATACTAATACGACTCACTATAGGGAGCCGCCACCATG GAC TAC AAG GAC GAC G
N-881 RV	TTTTTTTTTTTTTTTTTTTTTTTTTTTTTTCA TTT ATA TTG TAA GAT TCT A
N-662 RV	TTTTTTTTTTTTTTTTTTTTTTTTTTTTTTCA ATA CAA ATT TCC CTC TTT
N-498 RV	TTTTTTTTTTTTTTTTTTTTTTTTTTTTTTCA GTC GCT AAT GAT TCT TG
N-391 RV	TTTTTTTTTTTTTTTTTTTTTTTTTTTTTTCA CCT CAA TGC ATC TGA ACC ATA
N-313 RV	TTTTTTTTTTTTTTTTTTTTTTTTTTTTTTCA AAC GTT TTC AAC ATG TTC ACT AT
N-293 RV	TTTTTTTTTTTTTTTTTTTTTTTTTTTTTTCA ATT TGG AAC TAC GAC AGG AT
Arp8 662-876 FW	TCGATGCATACTAATACGACTCACTATAGGGAGCCGCCACCATG TGTG ATTTGAATGA CG
Arp8 662-876 RV	TTTTTTTTTTTTTTTTTTTTTTTTTTTTTTCA TTT ATA TTG TAA GAT TCT A
Arp8 498-662 FW	TCGATGCATACTAATACGACTCACTATAGGGAGCCGCCACCATG TGC GTA GTA AAT ATA G
Arp8 498-662 RV	TTTTTTTTTTTTTTTTTTTTTTTTTTTTTTCA ATA CAA ATT TCC CTC TTT
Arp4 peptide markers for CnBr cleavage	
Name	Sequence (5 ′ - 3 ′)
Arp4 N FW	TCGATGCATACTAATACGACTCACTATAGGGAGCCGCCACCATG TCCAATG CTGCTTTGC
N-489 RV	TTTTTTTTTTTTTTTTTTTTTTTTTTTTTTCA TCT AAA CCT ATC GTT AAG C
N-132 RV	TTTTTTTTTTTTTTTTTTTTTTTTTTTTTTCA CAT GCC TTC TAA GAG CAC
N-174 RV	TTTTTTTTTTTTTTTTTTTTTTTTTTTTTTCA CAT ACC ATC CAC TAT TGG

N-394 RV	TTTTTTTTTTTTTTTTTTTTTTTTTTTTTTTCA CAT TAT AGA CGA ATA AAC
N-425 RV	TTTTTTTTTTTTTTTTTTTTTTTTTTTTTTTCA CAT TAA CCT ATC ACT TAA TCC
Arp4 C RV	TTTTTTTTTTTTTTTTTTTTTTTTTTTTTTTCA TCT AAA CCT ATC GTT AAG C
132-C FW	TCGATGCATACTAATACGACTCACTATAGGGAGCCGCCACCATG CAAT TTGAAGCCTG TT
174-C FW	TCGATGCATACTAATACGACTCACTATAGGGAGCCGCCACCATG ACATTATC AAAGAGTA
394-C FW	TCGATGCATACTAATACGACTCACTATAGGGAGCCGCCACCATG AGCAGTGA TGTGGATC
425-C FW	TCGATGCATACTAATACGACTCACTATAGGGAGCCGCCACCATG ACAGA ACTAAACAAA A
Arp4 174-394 FW	TCGATGCATACTAATACGACTCACTATAGGGAGCCGCCACCATG ACATTATC AAAGAGTA
Arp4 174-394 RV	TTTTTTTTTTTTTTTTTTTTTTTTTTTTTTTCA CAT TAT AGA CGA ATA AAC
Arp4 132-425 FW	TCGATGCATACTAATACGACTCACTATAGGGAGCCGCCACCATG CAAT TTGAAGCCTG TT
Arp4 132-425 RV	TTTTTTTTTTTTTTTTTTTTTTTTTTTTTTTCA CAT TAA CCT ATC ACT TAA TCC
Arp4 peptide markers for NTCB cleavage	
Name	Sequence (5 ′ - 3 ′)
Arp4 N FW	TCGATGCATACTAATACGACTCACTATAGGGAGCCGCCACCATG TCCAATG CTGCTTTGC
N-154 RV	TTTTTTTTTTTTTTTTTTTTTTTTTTTTTTTCA ATT GGG TCT ACC TGC TGC
N-249 RV	TTTTTTTTTTTTTTTTTTTTTTTTTTTTTTTCA TAT ATG ACA AAG TGT TTC
Arp4 C RV	TTTTTTTTTTTTTTTTTTTTTTTTTTTTTTTCA TCT AAA CCT ATC GTT AA
136-C FW	TCGATGCATACTAATACGACTCACTATAGGGAGCCGCCACCATG TGT TAC TTA GCA CCC A
249-C FW	TCGATGCATACTAATACGACTCACTATAGGGAGCCGCCACCATG TGC CCAACAAAAA CTT
Arp4 136-249 FW	TCGATGCATACTAATACGACTCACTATAGGGAGCCGCCACCATG TGT TAC TTA GCA CCC A
Arp4 136-249 RV	TTTTTTTTTTTTTTTTTTTTTTTTTTTTTTTCA TAT ATG ACA AAG TGT TTC
